# COVID-19: What Is Next for Portugal?

**DOI:** 10.3389/fpubh.2020.00392

**Published:** 2020-08-21

**Authors:** Ahmed Nabil Shaaban, Barbara Peleteiro, Maria Rosario O. Martins

**Affiliations:** ^1^Global Health and Tropical Medicine (GHTM), Institute of Hygiene and Tropical Medicine (IHMT), NOVA University of Lisbon, Lisbon, Portugal; ^2^EPIUnit - Instituto de Saúde Pública, Universidade Do Porto, Porto, Portugal; ^3^Departamento de Ciências da Saúde Pública e Forenses e Educação Médica, Faculdade de Medicina da Universidade Do Porto, Porto, Portugal

**Keywords:** COVID-19, health inequalites, health system, quality indicators—healthcare, mental health, economic crisis

## Abstract

Highly infectious with the possibility of causing severe respiratory complications, the novel COVID-19 began stretching health systems beyond their capacity all over the world and pushing them to breaking points. Giving the devastating effects caused by this infection, unprecedented measures have to be adopted in order to mitigate its impacts on the health system. This perspective aims to review the epidemic of COVID-19 in Portugal, possible areas of improvement, and potential interventions that can help to mitigate the effect of COVID-19 on the Portuguese health system.

By June 3, 2020, the Severe Acute Respiratory Syndrome-Coronavirus-2 (SARS-CoV-2) has infected 33,261 individuals with 1,447 mortalities in Portugal (1). Unfortunately, this crisis came shortly after the recent recovery from the financial crisis that heavily affected the country in 2011, during which Portugal was obligated to sign-up for a bailout program from several funding entities, including the European Central Bank and the International Monetary Fund (2, 3). Accordingly, the country went through strict fiscal austerity that resulted in proposing unprecedented implementations of social expense cuts and continuous cuts to public expenditure on health care (2, 4, 5). Given the expectations of inevitable global recession due to COVID-19, which may surpass the global recession of 2009 to 2011 (6–8), it is expected that once again the health system in Portugal may become a target for cost containment in the long run. In general, and during economic crises, the health sector became vulnerable and a target for budget cuts owing to its size and the high potential for improved performance (3). Estimates regarding the economic impact of the COVID-19 in Portugal, if the crisis remains until mid-June, forecast GDP decline in 2020 of −6.9% (95% confidence interval: −9.2 to −4.6%) (7). These estimates predict Portugal to be among the most affected by the crisis in comparison to other countries such as Brazil, China, or the United States, owing to the high contribution of tourism to the Portuguese economy (7). We can understand from these estimates that, even if the current containment measures, namely, quarantines and social distancing, succeed in controlling the outbreak in Portugal, the economic implications of this crisis will affect the country in a post COVID-19 era. Some early results of the economic slowdown due to COVID-19 included a decline in the real estate market in regions with the greatest dynamism in the housing market and tourism, namely, the Lisbon Metropolitan Area and the Algarve (9). Moreover, the number of unemployed individuals registered in 74 municipalities during April 2020 was more than twice the registered number in the same month of the previous year (9). However, and unlike the financial crisis of 2011, any interventions or measures toward cost containment of the health sector should be taken with great precaution. In the one hand, any budget cuts that may affect the health sector in the future will limit the ability of the already exhausted sector in functioning against any recurrent outbreak, given the high risk of COVID-19 outbreaks over recurrent or seasonal waves (10–12). On the other hand, the economic situation of the country, in light of lower economic growth rates, may limit further spending on health. Accordingly, it is more important than ever to obtain an optimal balance between health and economic stability. This perspective aims to review possible flaws in the health sector and potential interventions which may help achieve this balance in Portugal. We also aim to provide measures that can help in mitigating the financial consequences of the COVID-19 on the health system and to provide recommendations that can contribute for containing any similar outbreak in the near future.

## COVID-19 Pandemic in Portugal

The first cases diagnosed with COVID-19 disease in Portugal were reported on March 2, 2020, while the first death was recorded on March 16, 2020 (13, 14). Portugal has adopted several measures in order to contain the transmission of the virus and contain the expansion of the disease. First, on March 18, 2020, the state of emergency was declared in Portugal, through the Decree of the President of the Republic No. 14-A/2020 (15). The decree imposed extraordinary urgent measures in the form of restrictions over domestic and international movements and the application of social distancing rules. Moreover, and due to the unprecedented health crisis imposed by the pandemic, the country had approved a new decree that allows legal immigrants with pending residence application who applied for legal residence in the country until March 18, when the state of emergency was decreed, to have access to health care services during the pandemic (16). With the measure, immigrants will have access to the same rights as Portuguese citizens, including use of the health system and social and financial support from the government. The decision also benefits those who have applied for asylum. Second, and regarding surveillance capabilities, and as of June 3, 2020, the government has set a network of testing centers that consists of 205 laboratories distributed across the country (17). Most of these laboratories follow the National Health Service (SNS) (45.2%) and the private sector (39.3%), but they also include other laboratories, namely, the military and the academic laboratories (15.7%) (18, 19). In April 2020, the average number of tests was 11,500 tests per day, and in May 2020, the average was 13,550 tests per day (20). As of June 3, 2020, more than 860,000 tests have been carried out to detect the disease in Portugal (20). About 40% of the COVID-19 tests were conducted in the Norte region of the country, followed by Lisbon and Vale do Tejo (25%) and the Centro (14%) regions (20). The remaining statistics are distributed over the remaining regions. Areas dedicated to treat patients with COVID-19 were created through several selected Emergency Service Units (ADC-SU) and COVID-19 Community Dedicated Areas (ADC-COMMUNITY) (21). The selection of COVID-19 dedicated areas depended on several factors that included population density, geographical dispersion, and the regional and local epidemiological evolution of COVID-19 (21).

As of June 3, 2020, the number of confirmed cases of COVID-19 per 10,000 inhabitants was 32.6 (9). Despite the progressive spread of the pandemic throughout the country, its spread continues to be characterized by a high regional heterogeneity and affected by various socio-economic impacts (9). However, analyzing the spread of COVID-19 by local administrative unit (LAU 1) (22), also known as municipality level, it translates into marked variation in the spread of the disease across municipalities. Portugal is divided into seven regions according to Nomenclature of Territorial Units for Statistics (NUTS II) (23) as follows: Norte, Centro, Lisbon Metropolitan Region (also known as Lisboa e Vale do Tejo), Alentejo, Algarve, Autonomous Region of the Azores, Autonomous Region of Madeira. The seven regions are divided in to 308 LAU 1 or municipalities. The Norte region carries a substantial burden especially when taking into account the absolute numbers of confirmed cases and deaths due to COVID-19. As of June 3, 2020, the confirmed cases in the Norte regions accounted for 50.5% of total confirmed cases and 55% of the total number of deaths (24) (see Table 1 for an informative overview of epidemiological situation in Portugal). At the municipality level, the number of confirmed cases per 10,000 inhabitants was higher than the national average in 50 municipalities (9). Of these, 31 were located in the Norte region, especially the municipalities located in the Metropolitan Area of Porto with more than 50 confirmed cases per 10,000 inhabitants, 11 municipalities in the Centro region, five in the Lisbon Metropolitan Region (the municipalities of Loures, Amadora, Lisbon, Odivelas, and Sintra), two in Alentejo region (the municipalities of Moura and Azambuja), and one municipality in the Autonomous Region of the Azores (the municipality of Nordeste) (9). Moreover, of the 50 municipalities with a number of confirmed cases per 10,000 inhabitants above the national average, 10 also had values of new confirmed cases per 10,000 inhabitants above the national average in which half of these municipalities were located in the Metropolitan Area of Lisbon (9).

**Table 1:** The Epidemic of COVID-19 in Portugal by Regions as of June 3, 2020.

**Portuguese Regions (NUTS II)**	**Norte**	**Centro**	**Lisbon**	**Alentejo**	**Algarve**	**Madeira**	**Azores**
Number of confirmed cases, *N* (%)	16,804 (50.5)	3,765 (11.3)	11,828 (35.6)	260 (0.8)	376 (1.1)	90 (0.3)	138 (0.4)
Number of deaths, *N* (%)	796 (55.0)	240 (16.6)	380 (26.3)	1 (0.1)	15 (1.0)	0 (0.0)	15 (1.0)
Number of laboratory testing centers, *N*	51	49	62	12	12	-	-
Number of dedicated COVID-19 centers, *N*	54	24	27	13	3	-	-
Number of municipalities above the national average of confirmed cases^a^, *N* (%)	31	11	5	2	0	0	1
Distribution of COVID-19 tests by region (%)	40	14	25	-	-	-	-

*Sources of data: Instituto Nacional de Estatística (INE) (9); Direção-Geral da Saúde (DGS) (1, 25); Servico National De Saude (SNS) (20)*.

*NUTS II Nomenclatura de Unidades Territoriais para Fins Estatísticos, nível II (Territorial Nomenclature Units for Statistical Purposes, level II); R.A. Região Autónoma (Autonomous Region)*.

a *Number of cases per 10,000 inhabitants*.

Moreover, 34 out of these 50 municipalities above the national level, almost two thirds, have a population density above the national average, and this highlights how population density can affect the spread of the disease (9). Of these 34 municipalities with population density above the national average, the highest number of confirmed cases per 10,000 inhabitants were recorded in the municipality of Ovar (123 cases per 10,000 inhabitants), while the lowest number were recorded in the municipality of Lisbon (52.1 cases per 10,000 inhabitants) (9).

## Measures to Mitigate the Effect of COVID-19 in Portugal

### Urgent Integration of Quality Indicators Within Hospitals Systems

Since we are encountering an unprecedented situation, immediate actions should be taken to preserve limited medical resources and prevent further unnecessary expenditure. Evidence from several countries suggest that unnecessary health spending, also known as wasteful spending, accounts for almost one-fifth of health expenditure in the form of unnecessary treatments or examinations, or health services provided with unnecessary higher costs (26, 27). Reducing or eliminating unnecessary health expenditure could be achieved without impairing quality of care (28). On the contrary, it will allow the health system to absorb an abrupt or unexpected increase in demand for medical resources, as in the case of COVID-19. As regards hospitals, hospitalizations or additional in-patient stays that consume a considerable amount of resources could be avoided with efficient treatment and management of chronic diseases, knowing that chronic diseases in Portugal consume a considerable amount of the health budget (29–34).

We pointed out, in previous contributions (30, 34), possible approaches to reduce the costs of healthcare in Portugal through integrating quality measures of hospitals' performance, namely thirty-day readmission rate and length of stay (LOS). Thirty-day hospital readmission is defined as an episode in which a patient is readmitted within 30 days from the last discharge. LOS is defined as the number of days a patient is hospitalized in relation to the admission diagnosis. High rates of thirty-day readmissions or unnecessary delayed discharge that contributes to higher LOS have been recognized as frequent and costly events (30, 35–37). For example, in the United States, one in five Medicare beneficiaries has a thirty-day readmission, with a cost of around $26 billion per year (37, 38). Accordingly, these measures have been widely used as a quality benchmark for health systems (30, 39–44). Given the expected implications of COVID-19 on the Portuguese economy and the health sector, it is mandatory that policymakers adopt these measures to impact cost and quality through payment incentives for hospitals or health care providers. By integrating quality indicators in the Portuguese health sector, we can focus on other areas of improvement, as listed in the following sections:

### Addressing Deficiencies in the Health System Infrastructure and Human Resources

The spread of COVID-19 created unprecedented pressure on hospitals and medical human resources, even in the most developed countries. With health system being stretched beyond its capacity, curative beds and critical care capacity require substantial review. Portugal has a total of 35,000 beds distributed between public, private, and public-private partnership hospitals; 22,400, 10,900, and 1,600, respectively (45). It is also important to mention that there was a decrease in the total number of beds over the period from 2007 to 2017 (45). For example, the total number of beds in 2017 was 84 beds lower than in 2016 and markedly lower than in 2007 with less 1,267 beds. This decline is owed to the steady increase in day surgery, the reinforcement of the long-term care networks, mergers between public hospitals and the closing of psychiatric hospitals (46, 47). Overall, Portugal has a lower number of curative beds per 100,000 population (325.2) compared to other European countries (6, 46).

The number of active physicians certified by the Portuguese Medical Association was 53,657 in 2018 (48). In addition, the number of active nurses certified by the Portuguese Nurses Association was 73,650 in 2018 (48). An increasing trend in the number of doctors and nurses have been reported in the period from 1960 to 2018 (48), while a decreasing trend in the number of inhabitants per doctor and nurses have been reported for the same period (49). However, these seemingly positive trends should be interpreted with caution. First, Portugal has one of the lowest ratios of nurses per 100,000 population (638 per 100,000 population) when compared with the European Union (EU) average (864 per 100,000 population) (46, 50). Second, the economic crisis of 2011 has led to significant outflows of emigration among doctors and nurses working in Portugal seeking better salaries and working conditions (46). For instance, the period from 2011 to 2015 witnessed the emigration of 1,631 doctors and 12,680 nurses from Portugal according to data from the Portuguese Medical and Nursing Associations (46). While current concerns about the shortage of medical human resources in Portugal are valid and real, what is more alarming is how this shortage can affect any strategies to curb the current infection. Moreover, we should expect that this pandemic will put the developed countries in a rival for attracting healthcare workers due to shortage in medical human resources or giving the crucial value they have had during this crisis. Accordingly, it is more important than ever that the Portuguese government set an action plan to retain the current work forces and address any further shortages. Moreover, since the density of the population plays an important role in shaping the distribution of COVID-19, solutions should be provided to ensure the allocation of medical resources to the municipalities with high population density.

### Addressing Health Inequalities in Portugal

Health inequalities can play an important role in shaping the distribution of COVID-19. Recent emerging data show the potential role of sex, race, and age on COVID-19 hospitalization and mortality rates, in which specific groups are disproportionately affected by the disease (51, 52). For example, the African-American community, which constitutes only 13% of the United States population, accounts for 33% of the hospitalizations related to COVID-19, while White Americans who constitute 76% of the total population account for 45% of the total hospitalizations (51). It is well-known that the African-American community in the United States carries a substantial burden when it comes to health inequalities with a higher risk of having a variety of health problems and less access to health care than White Americans (53–55). These findings are especially worrisome when considering how the apparent aspects of health inequalities can aggravate the COVID-19 distribution in Portugal. It is important to mention that socioeconomic characteristics are important indicators for health inequalities in Portugal (34, 56, 57). Portugal has a high proportion of elderly population, which is among the most affected by COVID-19, with those aged 65 years or more accounting for almost 20% of the total population (58). Table 2 shows the substantial effect of COVID-19 among the elderly population in Portugal in which infections among those aged above 60 years represent 32.7% of the total infections, while deaths among the same age group accounts for 95.4% of the deaths (1).

**Table 2:** Number of confirmed cases and deaths by age in Portugal as of June 3, 2020.

**Age Groups**	**Confirmed Cases**		**Deaths**	
	***N***	**(%)**	***N***	**(%)**
0–19	1,864	(5.6)	0	(0.0)
20–39	9,502	(28.6)	3	(0.2)
40–59	11,031	(33.2)	63	(4.3)
60–69	3,600	(10.8)	128	(8.8)
70–79	2,649	(8.0)	278	(19.2)
80+	4,615	(13.9)	975	(67.4)
Total	33,261	(100.0)	1,447	(100.0)

*Sources of data, Direção-Geral da Saúde (DGS) (1)*.

Migrants' health in Portugal illustrates another aspect of inequality, which translates into migrants using less and reporting more access restrictions (59). Although COVID-19 morbidities and fatalities by immigration status are not available yet, probably existing inequalities will be exacerbated in the present context. These expectations are supported by recent figures from the epidemiological bulletin of the Directorate-General for Health (DGS) indicating that municipalities located in the Metropolitan Area of Lisbon, which is characterized by having high migrants' concentrations, started to show a marked increase in the new cases per 10,000 inhabitants (1, 9). Over 50% of migrants are living in the Lisbon Metropolitan Area which is the home of 30% of the total Portuguese population (60). Also, it is important to know that municipalities with high concentrations of migrants record population density above the national level. For example, the municipality of Amadora, in the Metropolitan Area of Lisbon, which is known to have one of the largest migrant populations in the country, namely, in the neighborhood of The Bairro da Cova da Moura, is recording the highest population density in the entire country with almost 8000 inhabitants per square kilometer (59), in comparison to the average national population density of 111.5 inhabitants per square kilometer (61). Moreover, the same municipality of Amadora, is currently recording the highest number of new confirmed cases per 10,000 inhabitants above the national average (11.1 new cases per 10,000 inhabitants), followed by municipalities in the same Metropolitan Area of Lisbon as follows: Loures (10.0), Odivelas (7.4), Sintra (5.8), and Lisbon (4.9), which are also known to have high concentrations of migrants. Also, the health authorities were obligated to take drastic measures in the form of closing restaurants, cafés, and bars in one of the poorest migrants' social neighborhood in the country “Vale de Chícharos,” also known as “Bairro da Jamaica,” to contain the spread of an outbreak of new cases detected among residents (62). These findings are alarming, given the strong evidence that migrants and ethnic minorities specifically carry a substantial burden when it comes to infectious diseases owing to the lack of access to preventive health services and information (63). Moreover, previous studies showed migrants are among the most affected by infectious diseases and epidemics during economic crises due to worsening living conditions and lack of access to healthcare and treatment (64). These concerns highlight the consequences of measures that do not ensure the full entitlement of migrants in the health system. Since the government allowed documented migrants full access to health care services, solutions should also be provided to guarantee undocumented migrants full access to healthcare services without bearing any financial or legal consequences, especially in the light of the increasing number of new confirmed cases in areas with high migrant concentrations. Undocumented migrants in Portugal have limited healthcare entitlements compared to documented migrants (59). This unprecedented public health crisis due to COVID-19 should emphasize that the exclusion of any vulnerable populations from health care could halt the fight against the spread of infection.

Another aspect of health inequality is the unequal geographical distribution of health services and human resources for health in Portugal. In Portugal, human resources for health, health equipment, and supplies are concentrated in Lisbon and Porto, when compared to the country's remote areas (46, 47). Moreover, relatively younger populations are concentrated in the country's coastal regions, which are well-known to have higher socio-economic positions and better access to health care services compared to the rest of the country (47, 65). On the contrary, residents of remote areas, with lower socio-economic indicators, have poor geographical access to health services, which influences their ability to utilize health care services (47). These facts are supported by the heterogeneous spread of the disease over the country. For example, the majority of municipalities that recorded confirmed cases above the national level were lock land municipalities (40 municipalities) against only 10 costal municipalities (59). Our concern is that these aspects of inequalities will contribute to the spread of the disease in Portugal. These concerns demand interventions that guarantee a fair distribution of medical resources all over Portugal knowing that areas with relatively old Populations are more deprived of health services. Policies should also be developed to ensure the full and sustainable inclusion of migrants in the national health system without bearing any financial or legal consequences.

### Improving Mental Health Services

The increasing mortalities and morbidities due to COVID-19 made health care workers and general population to experience mental health problems such as depression and anxiety (66, 67). Moreover, the quarantine measures imposed to contain SARS-CoV-2 transmission that resulted in unprecedented social distancing and altered lifestyles began to have serious effects on mental health (68, 69). We might also expect (these associations tend to worsen) seeing similar effects as rates of unemployment, job loss, and poverty due to the economic effect of COVID-19 are increasing. For instance, during the economic crisis of 2011, Portugal witnessed a similar situation in which there was a surge in mental health problems (70, 71). In fact, the associations between the implications of economic crisis, such as unemployment or poverty, and mental health problems are well-documented (24).

These findings may be deemed worrying given the weaknesses and unpreparedness of the mental health services in Portugal to respond to such sharp demand. In the last decade, Portugal has witnessed a decrease in the number of psychiatric beds in favor of promoting community-based mental health services (28). However, a recent assessment of the Portuguese mental health plan indicated that country is still far from obtaining this goal (72). Also, it is important to know that that mental health in Portugal is lagging, compared to other European countries, in terms of the high prevalence of mental problems and the development of community-based mental health services (73, 74). Despite this fact, only a small proportion of patients who have mental illness have access to public specialized mental health services (73). In addition, mental health services in Portugal have substantial insufficiencies regarding equity and quality of care (73), given the substantial cost of mental health illness in EU in general, which is estimated to account for more than 4% of GDP (28), Portugal should put in place policies to address mental health among the population in general and to ensure emergency access to treatment for individuals affected by COVID-19 through establishing procedures for psychological crisis interventions.

### Preparedness Is the Key

If there is one lesson to be learned from the COVID-19 pandemic, it will be how to advance preparedness in other countries to mitigate the effect of the outbreak, and this should be instructive for Portugal. Taiwan and Singapore's response to the COVID-19 has been considered as a model, thanks to the SARS outbreak in 2013. These countries were among the most affected ones during the SARS outbreak (75–77). However, afterwards, they have established and developed their outbreak preparedness policies (75, 77). These policies included developing a public health action plan for facilitating rapid responses for the following crisis, holding regular exercises, establishing a central command center for epidemics, and building new infrastructures equipped with hundreds of negative-pressure isolation rooms and public health preparedness clinics (77, 78). As a result, they were able to successfully mitigate and contain the virus spread and keep it under control. Given this success and in light of the devastating implications of COVID-19, understanding and adopting the strategies implemented in these countries and their effectiveness may enlighten health policymakers in Portugal. As a starting point, an urgent public health response plan for allowing rapid actions for any possible future outbreak should be established in Portugal. This plan should include strategies to address shortages in human or medical resources or any flaws in the health system infrastructures. Hospitals also need guidelines to manage their spaces, human resources, and supplies to be able to contain any future similar outbreaks. Any plans should also consider reviewing the number and distribution of ventilators in the country, which is critical in treating severely ill patients. Moreover, specific specialties should be the focus of significant investment; for example, anesthesiologists, radiologists, and emergency room physicians should have particular skills that make them notably valuable to treat severely ill COVID-19 patients. The plan should also target the deficiencies in specialties such as public health doctors, which represent only 1.5% of the total active doctors in Portugal (46), and medical disaster specialists.

The health sector in Portugal can be more efficient in order to increase its ability to absorb any similar outbreaks by achieving that we can work on improving different aspects within the health sector by allocating resources, addressing shortage in medical and human resources. This unprecedented situation imposed the fact that public health cannot be achieved anymore without addressing health inequalities especially among migrant populations. The absence of a treatment or vaccine for COVID-19 should not be considered an excuse for the lack of national strategies and actions to contain and mitigate the effect of the current pandemic or any future similar outbreaks that pose an unprecedented threat to public health and the economy.

## Data Availability Statement

The original contributions presented in the study are included in the article/supplementary material, further inquiries can be directed to the corresponding author/s.

## Author Contributions

AS conceived the work, reviewed literature, and wrote the manuscript. BP and MM supervised the work and wrote the manuscript. All authors have agreed on the final version of the manuscript.

## Conflict of Interest

The authors declare that the research was conducted in the absence of any commercial or financial relationships that could be construed as a potential conflict of interest.

## References

[1] Direção-Geral da Saúde (DGS) Relatório de Situação n° 093. Available online at: https://covid19.min-saude.pt/wp-content/uploads/2020/06/93_DGS_boletim_20200603.pdf (accessed June 03, 2020).

[2] Sakellarides C Castelo-Branco L Barbosa P Azevedo H The Impact of the Financial Crisis on the Health System and Health in Portugal. Copenhagen: World Health Organization (2014).

[3] Baeten R Thomson S Health care policies: European debate and national reforms. In: *Social Developments in the European Union* Brussels: European Social Observatory (2011) 2012:187–212.

[4] Augusto GF. Cuts in Portugal's NHS could compromise care. Lancet. (2012) 379:400. 10.1016/s0140-6736(12)60174-3 22312642

[5] Karanikolos M Mladovsky P Cylus J Thomson S Basu S Stuckler D. Financial crisis, austerity, and health in Europe. Lancet. (2013) 381:1323–31. 10.1016/S0140-6736(13)60102-6 23541059

[6] Barua S Understanding coronanomics: the economic implications of the Coronavirus (COVID-19) pandemic. SSRN Elect J. (2020). Available online at: https://ssrn.com/abstract=3566477

[7] Fernandes N Economic effects of Coronavirus outbreak (COVID-19) on the world economy. SSRN Elect J. (2020). Available online at: https://ssrn.com/abstract=3557504

[8] Ruiz E Arturo M, Economic waves: the effect of the wuhan COVID-19 on the world economy (2019-2020). SSRN Elect J. (2020). Available online at: https://ssrn.com/abstract=3545758

[9] Instituto Nacional de Estatística (INE) Indicadores de contexto para a pandemia COVID-19 em Portugal. COVID-19: uma leitura territorial do contexto demográfico e do impacto socioeconómico - Dados até 03 de junho. (2020). Available online at: https://www.ine.pt/xportal/xmain?xpid=INE&xpgid=ine_destaques&DESTAQUESdest_boui=434670582&DESTAQUESmodo=2&xlang=pt (accessed June 5, 2020).

[10] Xu S Li Y. Beware of the second wave of COVID-19. The Lancet. (2020) 32277876 10.1016/S0140-6736(20)30845-X PMC7194658

[11] Leung K Wu JT Liu D Leung GM. First-wave COVID-19 transmissibility and severity in China outside Hubei after control measures, and second-wave scenario planning: a modelling impact assessment. The Lancet. (2020) 32277878 10.1016/S0140-6736(20)30746-7 PMC7195331

[12] Sajadi MM Habibzadeh P Vintzileos A Shokouhi S Miralles-Wilhelm F Amoroso A. Temperature, humidity and latitude analysis to predict potential spread and seasonality for COVID-19. SSRN Elect J. (2020). Available online at: https://ssrn.com/abstract=3550308 10.1001/jamanetworkopen.2020.11834 PMC7290414 32525550

[13] Direção-Geral da Saúde (DGS) Relatório de Situação n° 015. Available online at: https://www.dgs.pt/em-destaque/relatorio-de-situacao-n-015-17032020-pdf.aspx (accessed March 17, 2020).

[14] Direção-Geral da Saúde (DGS) Relatório de Situação n° 001. Available from: https://covid19.min-saude.pt/wp-content/uploads/2020/03/Relato%CC%81rio-de-Situac%CC%A7a%CC%83o-1.pdf (accessed March 03, 2020).

[15] Diário da República Decreto do Governo que regulamenta o estado de emergência. Decreto n.° 2-A/2020. Available online at: https://www.portugal.gov.pt/pt/gc22/comunicacao/documento?i=decreto-do-governo-que-regulamenta-o-estado-de-emergencia- (accessed March 18, 2020).

[16] Diário da República DESPACHO N.° 3863-B/2020 - DIÁRIO DA REPÚBLICA N.° 62/2020, 3° SUPLEMENTO, SÉRIE II DE. Available online at: https://dre.pt/documents/10184/2816226/Despacho_3863_B_2020_Translate.pdf/8abb060d-1858-45c3-852a-a5dd983fd1da (accessed March 27, 2020).

[17] Direção-Geral da Saúde (DGS) Laboratórios Referenciados. Available online at: https://covid19.min-saude.pt/laboratorios-referenciados/ (accessed June 7, 2020).

[18] Direção-Geral da Saúde (DGS) Portugal já realizou mais de 600 mil testes de diagnóstico à COVID-19. Available online at: https://covid19.min-saude.pt/portugal-ja-realizou-mais-de-600-mil-testes-de-diagnostico-a-covid-19/ (accessed June 3, 2020).

[19] Direção-Geral da Saúde (DGS) Portugal já fez mais de um milhão de testes de diagnóstico. Available online at: https://covid19.min-saude.pt/portugal-ja-fez-mais-de-um-milhao-de-testes-de-diagnostico/?fbclid=IwAR35DU5DiTASpnXg9S41S73Xa7rJSZWuosiMlO34baefHuH8qvvToEsRRK4 (accessed June 17, 2020).

[20] Servico National De Saude Covid-19 Testes de diagnóstico. Available online at: https://www.inem.pt/2020/06/04/covid-19-testes-de-diagnostico-2/ (accessed June 03, 2020).

[21] Direção-Geral da Saúde (DGS) Áreas Dedicadas COVID-19. Available online at: https://covid19.min-saude.pt/areas-dedicadas-covid-19/ (accessed June 7, 2020).

[22] European Commission Eurostat Local Administrative Units (LAU). Available online at: https://ec.europa.eu/eurostat/web/nuts/local-administrative-units (accessed May 30, 2020).

[23] European Commission Eurostat NUTS - Nomenclature of Territorial Units. Available online at: https://ec.europa.eu/eurostat/web/nuts/background (accessed May 30, 2020).

[24] World Health Organization Impact of Economic Crises on Mental Health. Copenhage: World Health Organization, Regional Office for European Union (2011).

[25] Direção-Geral da Saúde (DGS) Ponto de Situação Atual em Portugal. Available online at: https://covid19.min-saude.pt/ (accessed June 10, 2020).

[26] Limb M A Fifth of Healthcare Spending is Wasted, Says OECD Report. London: British Medical Journal Publishing Group (2017). 10.1136/bmj.j215 28087592

[27] OECD/EU Health at a Glance: Europe 2018: State of Health in the EU Cycle. Paris: OECD Publishing (2018).

[28] Caldas de Almeida J Mateus P Tomé G Joint Action on Mental Health and Well-Being Towards Community-Based and Socially Inclusive Mental Health Care. (2015) Available online at: https://ec.europa.eu/health/sites/health/files/mental_health/docs/2017_towardsmhcare_en.pdf (accessed March 26, 2020).

[29] Lopes JM Gonçalves FR Borges M Redondo P Laranja-Pontes J. The cost of cancer treatment in Portugal. Ecancermedicalscience. (2017) 11:765. 10.3332/ecancer.2017.765 28955401 PMC5606294

[30] Shaaban AN Martins O Rosario M. The importance of improving the quality of care among HIV/AIDS hospitalizations in Portugal. Front Public Health. (2019) 7:266. 10.3389/fpubh.2019.00266 31572706 PMC6753230

[31] Ferreira de Magalhães M Amaral R Pereira AM Sá-Sousa A Azevedo I Azevedo LF. Cost of asthma in children: a nationwide, population-based, cost-of-illness study. Pediatr Allergy Immunol. (2017) 28:683–91. 10.1111/pai.12772 28815837

[32] Barbosa J Ferreira-Magalhães M Sá-Sousa A Azevedo L Fonseca J. Cost of asthma in Portuguese adults: a population-based, cost-of-illness study. Rev Port Pneumol. (2017) 23:323–30. 10.1016/j.rppnen.2017.07.003 28807558

[33] Fiorentino F Ascenção R Gouveia M Costa J Broeiro P Fonseca C The cost of illness of heart failure in Portugal. Value Health. (2017) 20:A610 10.1016/j.jval.2017.08.1203

[34] Shaaban AN Dias SS Muggli Z Peleteiro B Martins MRO. Risk of readmission among HIV patients in public portuguese hospitals: longitudinal multilevel population-based study. Front Public Health. (2020) 8:15. 10.3389/fpubh.2020.00015 32154201 PMC7049668

[35] Horwitz L Partovian C Lin Z Herrin J Grady J Conover M Hospital-Wide (All-Condition) 30-Day Risk-Standardized Readmission Measure. New Haven, CT (2011). Available online at: http://146.123.140.205/Medicare/Quality-Initiatives-Patient-Assessment-Instruments/MMS/downloads/MMSHospital-WideAll-ConditionReadmissionRate.pdf (accessed October 27, 2013)

[36] Joynt KE Jha AK. Thirty-day readmissions—truth and consequences. New Engl J Med. (2012) 366:1366–9. 10.1056/NEJMp1201598 22455752

[37] Leppin AL Gionfriddo MR Kessler M Brito JP Mair FS Gallacher K. Preventing 30-day hospital readmissions: a systematic review and meta-analysis of randomized trials. JAMA Intern Med. (2014) 174:1095–107. 10.1001/jamainternmed.2014.1608 24820131 PMC4249925

[38] Jencks SF Williams MV Coleman EA. Rehospitalizations among patients in the Medicare fee-for-service program. New Engl J Med. (2009) 360:1418–28. 10.1056/NEJMsa0803563 19339721

[39] Nijhawan AE Kitchell E Etherton SS Duarte P Halm EA Jain MK. Half of 30-day hospital readmissions among HIV-infected patients are potentially preventable. AIDS Patient Care and STDs. (2015) 29:465–73. 10.1089/apc.2015.0096 26154066 PMC4564018

[40] Coelho LE Ribeiro SR Japiassu AM Moreira RI Lara PC Veloso VG. Thirty-day Readmission Rates in an HIV-infected Cohort From Rio de Janeiro, Brazil. J Acquir Immune Defic Syndr. (2017) 75:e90–e8. 10.1097/QAI.0000000000001352 28291051 PMC5484736

[41] Berry S Fleishman J Moore R Gebo K. Thirty-day hospital readmissions for adults with and without HIV infection. HIV Med. (2016) 17:167–77. 10.1111/hiv.12287 26176492 PMC4713370

[42] Boccuti C Casillas G Aiming for Fewer Hospital U-turns: The Medicare Hospital Readmission Reduction Program. The Henry J. Kaiser Family Foundation (2015). Available online at: http://slcsuperiorhomecare.com/wp-content/uploads/2015/06/Kaiser-Readmission-paper.pdf (accessed April 2, 2020).

[43] Brasel KJ Lim HJ Nirula R Weigelt JA. Length of stay: an appropriate quality measure? Arch Surg. (2007) 142:461–6. 10.1001/archsurg.142.5.461 17515488

[44] Kahn KL Keeler EB Sherwood MJ Rogers WH Draper D Bentow SS. Comparing outcomes of care before and after implementation of the DRG-based prospective payment system. JAMA. (1990) 264:1984–8. 10.1001/jama.1990.03450150084036 2120477

[45] Instituto Nacional de Estatística - Estatísticas da Saúde 2017 Lisboa: INE (2019). Available online at: https://www.ine.pt/xurl/pub/320460040>.ISSN_2163-1637.ISBN978-989-25-0482-7 (accessed March 17, 2020).

[46] Simões J Augusto GF Fronteira I Hernández-Quevedo C Portugal: health system review. Health Syst Trans. (2017) 19:1–184. Available on line at: http://www.euro.who.int/__data/assets/pdf_file/0007/337471/HiT-Portugal.pdf?ua=1 28485714

[47] OECD/European Observatory on Health Systems and Policies (2017), Portugal: Country Health Profile 2017, State of Health in the EU, OECD Publishing, Paris/European Observatory on Health Systems and Policies, Brussels.

[48] PORDATA Healthcare Personnel: Doctors, Dentists, Odontologists, Nurses and Pharmacists. Available online at: https://www.pordata.pt/en/Portugal/Healthcare+personnel+doctors++dentists++odontologists++nurses+and+pharmacists-144 (accessed Apr 11, 2020).

[49] PORDATA Number of Inhabitants Per Doctor and Healthcare Personnel. Available from: https://www.pordata.pt/en/Portugal/Number+of+inhabitants+per+doctor+and+healthcare+personnel-640 (accessed Apr 11, 2020).

[50] Eurostat Healthcare Personnel Statistics - Nursing and Caring Professionals. Available online at: https://ec.europa.eu/eurostat/statistics-explained/index.php?title=Healthcare_personnel_statistics_-_nursing_and_caring_professionals&oldid=452027#Healthcare_personnel_.E2.80.94_nursing_professionals (accessed Apr 11, 2020).

[51] Garg S Hospitalization Rates and Characteristics of Patients Hospitalized with Laboratory-Confirmed Coronavirus Disease 2019—COVID-NET, 14 States, March 1–30, (2020). MMWR Morbidity and Mortality Weekly Report. Atlanta, GA (2020) 69. 10.15585/mmwr.mm6915e3 PMC7755063 32298251

[52] Wu Z McGoogan JM. Characteristics of and important lessons from the coronavirus disease 2019 (COVID-19) outbreak in China: summary of a report of 72 314 cases from the Chinese Center for Disease Control and Prevention. JAMA. (2020) 323:1239–42. 10.1001/jama.2020.2648 32091533

[53] Dressler WW Health in the African American community: accounting for health inequalities. Med Anthropol Quarterly. (1993) 7:325–45. 10.1525/maq.1993.7.4.02a00030

[54] Schulz A Israel B Williams D Parker E Becker A James S. Social inequalities, stressors and self reported health status among African American and white women in the Detroit metropolitan area. Social Sci Med. (2000) 51:1639–53. 10.1016/s0277-9536(00)00084-8 11072884

[55] Jackson PB. Health inequalities among minority populations. J Gerontol Series B. (2005) 60:S63–7. 10.1093/geronb/60.Special_Issue_2.S63 16251593

[56] Santana P Acessibilidade e utilizaçao dos serviços de saúde. Ensaio metodológico em economia da saúde. Coimbra: Faculdade de Letras da Universidade de Coimbra Universidade de Coimbra (1993)

[57] Giraldes MdR Morbilidade declarada no INS 1995/96. Que respostas?–Uma abordagem realizada numa perspectiva de equidade. Lisbon: Revista Portuguesa de Sáude Pública (1998) 16:43–60.

[58] PORTUGAL REPORT United Nations Economic Commission for Europe (UNECE) Third Review and Appraisal of the Regional Implementation Strategy (RIS) of the Madrid International Plan of Action on Ageing (MIPPA). (2017) Available online at: https://www.unece.org/fileadmin/DAM/pau/age/country_rpts/2017/POR_report_EN.pdf (accessed April 1, 2020).

[59] Shaaban AN Morais S Peleteiro B. Healthcare services utilization among migrants in Portugal: results from the National Health Survey 2014. Journal of immigrant and minority health. (2018) 21:219–29. 10.1007/s10903-018-0744-3 29644552

[60] Migration Integration Policy Index Health Strand (MIPEX) Country Report Portugal. Brussels: International Organization for Migration (2015).

[61] Pordata O seu município em números. Available online at: https://www.pordata.pt/Municipios/Quadro+Resumo/%c3%81rea+Metropolitana+do+Porto+(NUTS+III)-251510 (accessed June 5, 2020).

[62] Publico Portugal Covid-19: Encerrados Oito Estabelecimentos no BAIRRO da Jamaica no Seixal. Available online at: https://www.publico.pt/2020/05/30/sociedade/noticia/covid19-encerrados-oito-estabelecimentos-bairro-jamaica-seixal-1918808 (accessed May 30, 2020).

[63] Lancet T Migration and Health: A Complex Relation. London: Elsevier (2006). 10.1016/S0140-6736(06)69423-3 16997643

[64] Ayuso-Mateos JL Barros PP Gusmão R. Financial crisis, austerity, and health in Europe. Lancet. (2013) 382:391–2. 23911370 10.1016/S0140-6736(13)61663-3

[65] Oliveira MD Bevan G. Measuring geographic inequities in the Portuguese health care system: an estimation of hospital care needs. Health Policy. (2003) 66:277–93. 10.1016/S0168-8510(03)00118-0 14637012

[66] Kang L Li Y Hu S Chen M Yang C Yang BX. The mental health of medical workers in Wuhan, China dealing with the 2019 novel coronavirus. Lancet Psychiatry. (2020) 7:e14. 10.1016/S2215-0366(20)30047-X 32035030 PMC7129673

[67] Xiang Y-T Yang Y Li W Zhang L Zhang Q Cheung T. Timely mental health care for the 2019 novel coronavirus outbreak is urgently needed. Lancet Psychiatry. (2020) 7:228–9. 10.1016/S2215-0366(20)30046-8 32032543 PMC7128153

[68] Brooks SK Webster RK Smith LE Woodland L Wessely S Greenberg N. The psychological impact of quarantine and how to reduce it: rapid review of the evidence. Lancet. (2020) 395:912–20. 10.1016/S0140-6736(20)30460-8 32112714 PMC7158942

[69] Venkatesh A Edirappuli S. Social distancing in covid-19: what are the mental health implications? BMJ. (2020) 369:m1379. 10.1136/bmj.m1379 32253182

[70] Augusto GF. Mental health in Portugal in times of austerity. Lancet Psychiatry. (2014) 1:109–10. 10.1016/S2215-0366(14)70251-2 26360565

[71] Santos JC Cutliffe J The recent global socioeconomic crisis and its effects on mental health in Portugal. Ment. Health Nurs. (2013) 33:33-5.

[72] Comissão Técnica de Acompanhamento da Reforma da Saúde Mental Relatório da Avaliação do Plano Nacional de Saúde mental 2007–2016 e propostas prioritárias para a extensão a 2020. Lisbon: Serviço Nacional de Saúde (2017). Available online at: https://www.sns.gov.pt/wp-content/uploads/2017/08/RelAvPNSM2017.pdf (accessed March 26, 2020).

[73] Caldas de Almeida JM. Portuguese National Mental Health Plan (2007-2016) executive summary. Ment Health Fam Med. (2009) 6:233–44. 22477915 PMC2873880

[74] Caldas de Almeida J Mateus P Tomé G Joint Action on Mental Health and Well-Being Towards Community-Based and Socially Inclusive Mental Health Care. Lisbon: Europe Union Reports (2015).

[75] Chen K-T Twu S-J Chang H-L Wu Y-C Chen C-T Lin T-H. SARS in Taiwan: an overview and lessons learned. Int J Infect Dis. (2005) 9:77–85. 10.1016/j.ijid.2004.04.015 15708322 PMC7110635

[76] Wong J Goh QY Tan Z Lie SA Tay YC Ng SY. Preparing for a COVID-19 pandemic: a review of operating room outbreak response measures in a large tertiary hospital in Singapore. Can J Anaesth. (2020) 67:732–45. 10.1007/s12630-020-01620-9 32162212 PMC7090449

[77] Liew MF Siow WT MacLaren G See KC. Preparing for COVID-19: early experience from an intensive care unit in Singapore. Critical Care. (2020) 24:83. 10.1186/s13054-020-2814-x 32151274 PMC7063757

[78] Wang CJ Ng CY Brook RH. Response to COVID-19 in Taiwan: big data analytics, new technology, and proactive testing. JAMA. (2020) 323:1327–1420. 10.1001/jama.2020.3151 32125371

